# Remarks on Schools of Instruction for Military and Naval Surgeons, in a Letter to Sir Robert Peel

**Published:** 1844-01

**Authors:** 


ART. VI.—*Remarks on Schools of Instruction for Military and Naval Surgeons, in a Letter to Sir Robert Peel.* By SIR GEORGE BALLINGHALL, M.D., Regius Professor of Military Surgery in the University of Edinburgh. 8vo, pp. 16.

THE praiseworthy object of this Letter is to point out the importance of having a professorship of military surgery in London and Dublin as well as in Edinburgh. Sir George's motives in making this proposal are singularly disinterested, and he argues his case zealously and well. We cannot help entertaining doubts, however, whether the institution of the chairs in question is necessary. The arguments derived from their existence and utility in the continental states, appear to us less valid than to Sir George. The extremely low state of the full-pay and half-pay of the medical officers in the continental armies, prevents fully-educated men from entering them, and interferes with the prosecution of study in the intervals of active service. The opposite state of things exists in this country. We willingly admit, however, that the military chair at Edinburgh has been very useful, especially since filled by Sir George; and it would give us pleasure to see his wishes fulfilled, and men of equal talents with himself appointed to instruct their junior brethren when on half-pay, in the other capital cities of the kingdom.

